# In Situ Localization of *Staphylococcus shinii* and *Staphylococcus succinus* in Infected *Rhipicephalus microplus* Ticks: Implications for Biocontrol Strategies

**DOI:** 10.3390/pathogens13121025

**Published:** 2024-11-21

**Authors:** Cesar A. Arreguin-Perez, Raquel Cossio-Bayugar, Hugo Aguilar-Diaz, Estefan Miranda-Miranda

**Affiliations:** Centro Nacional de Investigación Disciplinaria en Salud Animal e Inocuidad, Instituto Nacional de Investigaciones Forestales Agrícolas y Pecuarias INIFAP, Boulevard Cuauhnahuac 8534, Jiutepec 62574, Morelos, Mexicoaguilar.hugo@inifap.gob.mx (H.A.-D.); miranda.estefhan@inifap.gob.mx (E.M.-M.)

**Keywords:** vestibular vagina, Gené’s organ, salivary glands, hemolymph, entomopathogenic bacteria

## Abstract

*Rhipicephalus microplus* is a blood-sucking parasite that causes heavy infestations on cattle and is a vector for severe tick-borne diseases, such as anaplasmosis and babesiosis, and poses a significant threat to the cattle industry. Cattle ticks show increasing acaricide resistance, which creates an additional problem concerning the inefficient chemical control of tick populations in cattle-grazing areas, necessitating the exploration of alternative tick biocontrol methods. Our study aimed to demonstrate the acaropathogenic efficacy of two bacterial species during experimental infections on *R. microplus*. Our experimental data confirmed that *S. shinii* and *S. succinus* exhibited significant acaropathogenic properties against *R. microplus,* as demonstrated by the tracking of fluorescent-labeled bacteria within the engorged-tick body. Our experiments revealed that both bacterial species could infect the hemolymph, salivary glands, and vestibular vagina of the tick, inducing histological changes in the affected organs that may impair feeding as well as reproductive capabilities. Gené’s organ infection was detected only in *S. succinus*. Our findings offer valuable insights for developing biocontrol strategies to manage *Rhipicephalus microplus* populations effectively.

## 1. Introduction

*Rhipicephalus microplus*, commonly known as the cattle tick, is a persistent bovine ectoparasite in tropical and subtropical cattle-grazing areas worldwide [1]. The lifecycle of this tick includes a nonparasitic larval phase on the ground [2] and a parasitic phase on the cattle that may cause direct harm to the host, leading to skin damage, anemia, weight loss, irritability, immunosuppression, and reduced milk production [3]. Moreover, during this parasitic phase, *R. microplus* may also act as a vector for diseases such as anaplasmosis and babesiosis [4,5], causing billions of dollars of damage to the cattle, milk, and leather industries worldwide [1].

The emergence of acaricidal resistance in ticks has prompted a global initiative to discover novel organisms that are pathogenic to ticks. This approach aims to achieve enhanced and environmentally sustainable management of *R. microplus* and other tick infestations in livestock, domestic animals, and humans [6,7,8,9]. Although research focusing on fungal infections in cattle ticks has been predominant in this field [6,8,10,11,12], the potential of bacterial infections in reducing *R. microplus* populations has been recognized recently [13,14,15]. The key acaropathogenic bacterial species include *Serratia* sp. [16], *Staphylococcus saprophyticus* [9], which was later reclassified as *S. shinii* by more stringent genomic studies [14], *S. xylosus* [14], *S. succinus* [14], and *Bacillus thuringiensis* [13]. All of these bacterial species have been demonstrated to be effective at controlling tick populations through experimental infections at several developmental stages of the cattle tick [13,14,16]. These bacterial infections cause pathogenic signs, including reduced oviposition in adult ticks, reduced larval hatching, and survival [13,14,16]. The pathogenic signs of bacterial infection reported in these studies include cuticle darkening, engorged female swelling, hypostome exudates, egg darkening, reduced oviposition, and oviposition of dry eggs [14].

When ticks act as vectors of infectious diseases, they can carry various microorganisms in their internal organs, such as *Anaplasma marginale* [17,18], *Borrelia burgdorferi* [19], *Ehrlichia canis* [20], and *Babesia* sp. [21]. Many tick pathogens follow a common infection sequence: the pathogen moves from the midgut to the salivary glands via the hemolymph. However, *Babesia* spp. is an exception, undergoing a transovarial transmission phase. Nevertheless, *Babesia* sp. also uses the salivary glands to continue its life cycle [21].

*Rhipicephalus microplus* is a blood-feeding parasite with a midgut that undergoes notable changes as feeding progresses [22]. Initially, it starts as a “small tube-like structure” that expands and differentiates [22,23]. This organ is integral to the energy metabolism of ticks [24] and serves as a crucial site for the lifecycle of hemoparasites such as *Babesia* and *Anaplasma* species [18,21,25].

Numerous microbiome analysis reports have documented the presence of bacteria from the *Staphylococcus* genus in various organs of the tick species *R. microplus* and other hard ticks. These organs include the salivary glands [26,27], gut [26,28], eggs [28], and ovaries of *Ixodes ricinus* [29]. The identified species, *S. pyogenes*, *S. aureus*, and *S. albus*, have been found in ticks [30]. On the other hand, reports indicate that staphylococcal infections in *R. microplus* can lead to increased mortality, reduced oviposition, and inhibition of the reproductive index [9,14]. However, information regarding the presence of the *Staphylococcus* genus in the ovaries of *R. microplus* is lacking [28,29].

This study aimed to locate *Staphylococcus shinii* and *S. succinus* infections in *R. microplus* ticks, determine the specific organs targeted by these bacteria, and understand their pathogenic activity.

## 2. Materials and Methods

### 2.1. Tick Rearing

The ‘Media Joya’ strain, known for its susceptibility to pesticides, was selected for this study [31]. The strain was initially propagated by infesting healthy, stable bovines with 20,000 larvae each. After 21 days, 75 semiengorged females, which ranged from 100–200 mg, were gathered and specifically chosen on the basis of their weight [31]. To ensure the absence of signs of infection, such as color changes and exudate presence, as well as the integrity of the hypostome, the ticks were washed with 0.1% benzalkonium chloride and 0.05% sodium nitrite for 10 min, followed by rinsing in distilled water for an additional 10 min as previously reported [14].

### 2.2. Bacterial Culture and Staining

*Staphylococcus shinni* S-1 [14] and *Staphylococcus succinus* [14] are nonmodified strains registered with the World Federation Culture Collection as CM-CNRG TB 100 and CM-CNRG TB 102, respectively. These strains are associated with the bioproject number PRJNA421192 in the GenBank database. *S. shinni* S-1 has the biosample identifier SAMN08134547 and the accession number GCA_002836805.1 (GenBank GCF_002836805.1), whereas *S. succinus* is identified by SAMN08134550 with the accession number GCF_002836835.1 in the GenBank database. *Escherichia coli*. Top 10 is a commercially available strain primarily used for plasmid cloning and transformation and was obtained from Invitrogen (Groningen, The Netherlands). This strain has been specifically modified for these purposes. Its genotype is F-mcrA (mrr-hsdRMS-mcrBC) ϕ80lacZΔM15 lacX74 recA1 araD139 (ara-leu)7697 galU galK rpsL (StrR) endA1 nupG, reflecting these specialized adaptations.

The bacterial strains *S. shinii* S-1, *S. succinus*, and *E. coli* Top 10 were cultivated in preprepared tryptic soy broth (STA; Sigma-Aldrich, Burlington, MA, USA) at 37 °C with 150 rpm agitation for 24 h. The bacterial cultures were subsequently adjusted to a concentration of 1 × 10^8^ colony-forming units (CFU)/mL in 10 mL of 0.1 M NaHCO_3_ (Sigma-Aldrich, Burlington, MA, USA) in phosphate-buffered saline (PBS; Sigma-Aldrich, Burlington, MA, USA) at pH 8. The bacteria were stained with 20 mg of fluorescein 5(6)-isothiocyanate (FITC; Sigma-Aldrich, Burlington, MA, USA) per strain and incubated for 30 min at 25 °C with continuous agitation. After incubation, the bacteria were centrifuged at 2000 rpm for 5 min and washed with PBS six times. Finally, the bacteria were resuspended in 10 mL of PBS and stored in a dark environment until further use.

### 2.3. Modified Adult Immersion Test

A modified adult immersion test (AIT) was used to evaluate the pathogenic activity of *S. shinii* S-1, *S. succinus*, and *E. coli* Top 10 against female *R. microplus* ticks [14]. Eighteen semiengorged ticks were immersed in PBS containing stained bacteria from *S. shinii*, *S. succinus*, and *E. coli* Top 10 at a concentration of 1 × 10^8^ colony-forming units (CFU)/mL. An additional control group was treated with PBS alone. After a 10-min exposure, the ticks were dried and incubated in separate Petri dishes for 24 and 48 h at 28 °C with 80% relative humidity. The samples were subsequently dissected for further analysis.

### 2.4. Tick Dissection, Hemolymph Extraction, and Epifluorescence Microscopy

To accurately locate the bacteria within *Rhipicephalus microplus*, the ovaries, Gené’s organs, salivary glands, vestibular vaginas, midgut, and tracheas of the treated semi-engorged females were meticulously dissected at 2-, 24-, and 48-h posttreatment, following well-established protocols [32]. The hemolymph was obtained by hypocuticular punctures, as described previously [33]. After dissection, the organs were preserved in PBS at −70 °C for examination. The organs were subsequently examined via epifluorescence microscopy (Axioscope 40, Carl Zeiss, Jena, Germany) equipped with FITC (Filter 09 Zeiss BP 450–490, FT 510, LP 515) and rhodamine (Filter 15 Zeiss BP 546/12, FT 580, LP 590) filters to identify the presence of infected organs and evaluate any damage caused by the infection.

## 3. Results

### 3.1. Experimental Bacterial Infection of Engorged Female Ticks

**Figure 1:**
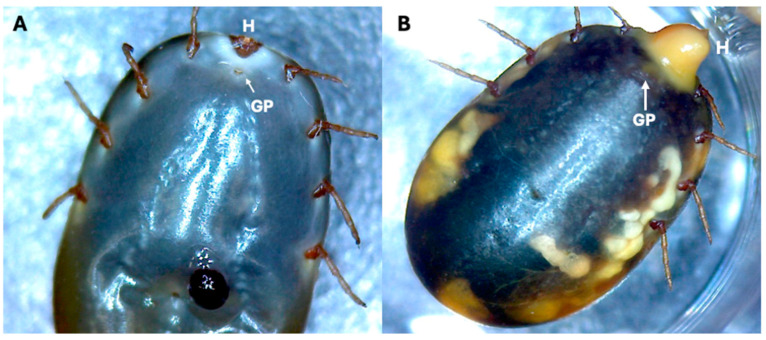
Signs of *Staphylococcus* infection in *Rhipicephalus microplus*-engorged females. During our experimental assessment of *Staphylococcus* infection in cattle ticks, we identified the main features of bacterial infection in engorged female ticks. (**A**). Uninfected female tick showing the positions of the genital pore (GP) and hypostome (H). (**B**). *Staphylococcus shinii*-infected female ticks showing signs of exudates covering both the genital pore (GP) and the hypostome (H). Other signs of Staphylococcus infection, in addition to exudate, include swollen bodies and color changes.

Fifteen days after infection, the engorged females displayed swelling and exudate in the genital orifice-hypostome area, as shown in Figure 1B. To determine the localization of the bacteria within the organs, infected ticks were examined at 2, 24, and 48 h post-infection to gain insight into the initial stages of the infection process.

### 3.2. Bacterial Organ Localization

**Figure 2:**
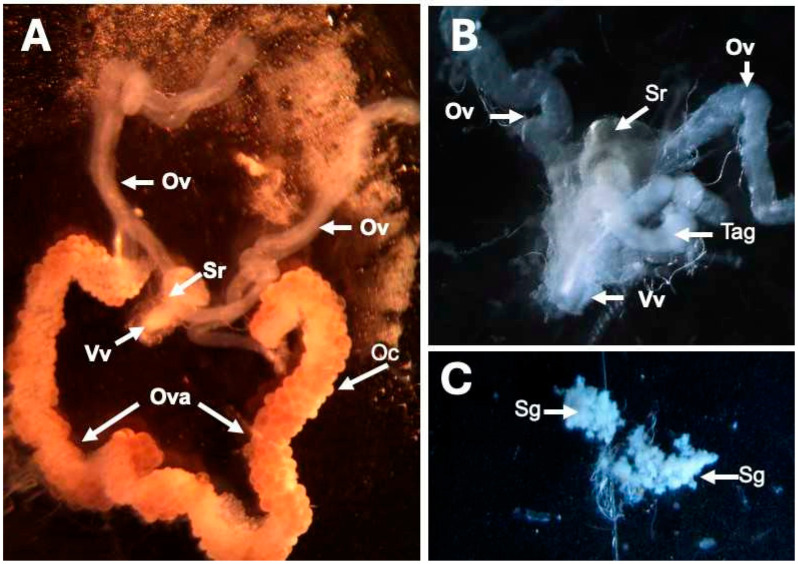
Dissections of adult female tick organs. (**A**) The female reproductive system at 20× magnification shows oviducts (Ov)., vestibular vagina (Vv), ovary (Ova), and seminal receptacle (Sr). (**B**) Close-up view of the vestibular vagina (Vv), seminal receptacle (Sr), oviducts (Ov), and tubular accessory gland (Tag) at 50× magnification. (**C**) Salivary glands (Sg) at 50× magnification.

Figure 2 shows the organ dissection of adult female ticks. Panel A illustrates the reproductive system, highlighting how the oviducts connect to a single ovary on each side (Ov). The oviducts also link to the vestibular vagina (Vv) and the seminal receptacle (Sr), which opens to the exterior through the genital pore (Figure 1 GP) [34]. Panel B provides a detailed view of the vestibular vagina (Vv), whereas panel C focuses on the salivary glands (Sg).

**Figure 3:**
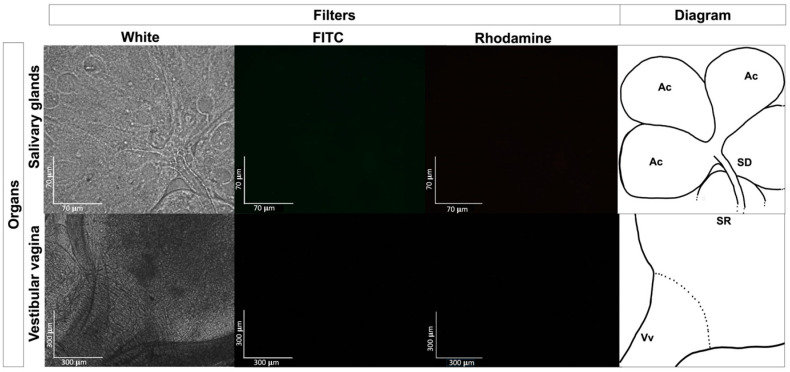
Experimental exposure of fluorescent *E. coli* and background autofluorescent control. Fluorescence microscopy images displaying *Rhipicephalus microplus* salivary glands and Gené’s organ. Images of tissue samples from the tick-treated group with 1 × 10^8^ UFC/mL *E. coli* at 24 h posttreatment were obtained via epifluorescence microscope. A schematic diagram was made to illustrate the salivary gland, acini, and vestibular vagina with the seminar receptacle. Images were captured using white, FITC, and rhodamine filters at 400× magnification. Ac: Acinni; SD: Salivary Duct; SR: Seminal receptacle; Vv: Vestibular vagina.

**Figure 4:**
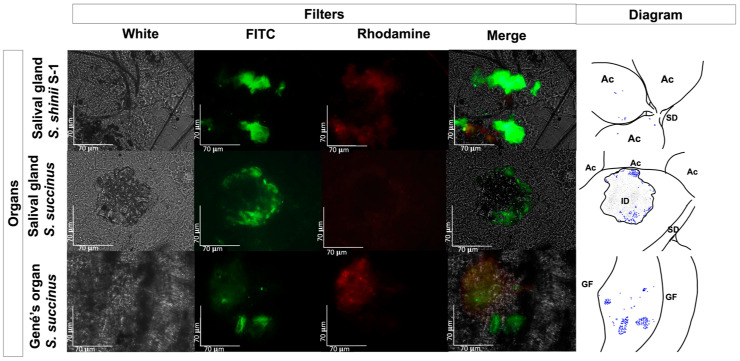
Fluorescence microscopy images displaying *Rhipicephalus microplus* salivary glands and Gené’s organ. Images of tissue samples from the tick-treated (group treated) with 1 × 10^8^ UFC/mL *S. succinus* or *S. shinii* S-1 24 h posttreatment were obtained via an epifluorescence microscope, and a schematic diagram was generated to illustrate the fluorescent bacteria in blue within the salivary and Gené’s organ. Images were captured using white, FITC, and rhodamine filters at 400× magnification. Ac: Acinni; SD: salivary duct; ID: infection damage; GF: Gené’s organ fold.

**Figure 5:**
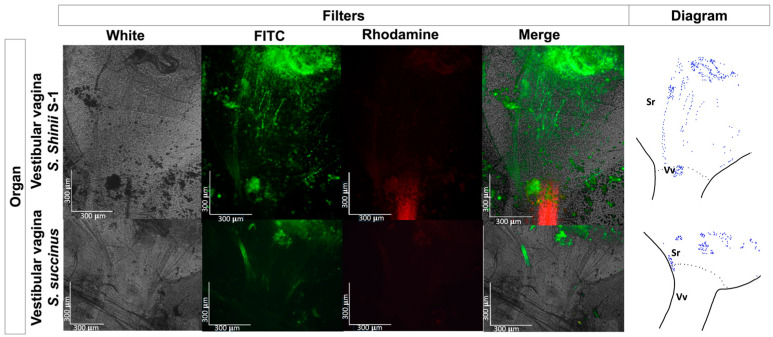
Fluorescence microscopy images of the vestibular vagina of *Rhipicephalus microplus*. Tissue samples from the tick group treated with 1 × 10^8^ UFC/mL *S. succinus* and *S. shinii* were captured via an epifluorescence microscope at 2- and 24-h posttreatment, and a schematic diagram was generated to illustrate the fluorescent bacteria in blue within the vestibular vagina. Images were captured using white, FITC, and rhodamine filters at 400× magnification. Sr: seminal receptacle; Vv: vestibular vagina.

**Figure 6:**
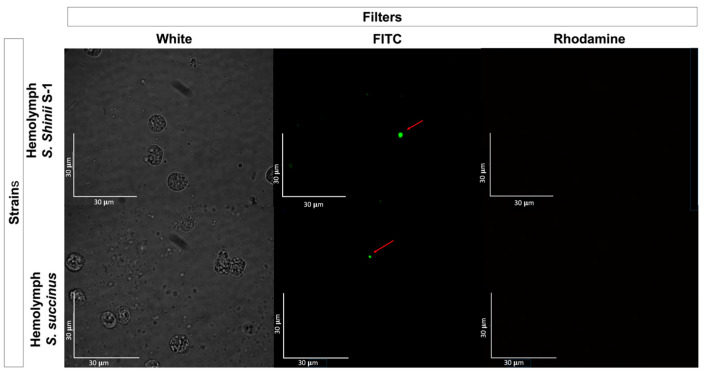
Fluorescence microscopy images showing the *Rhipicephalus microplus* hemolymph from the group treated with 1 × 10^8^ UFC/mL *S. succinus-shinii* at 2 h posttreatment. The arrows indicate the stained bacteria in the image. Images were captured using white, FITC, and rhodamine filters at 1000× magnification.

**Figure 7:**
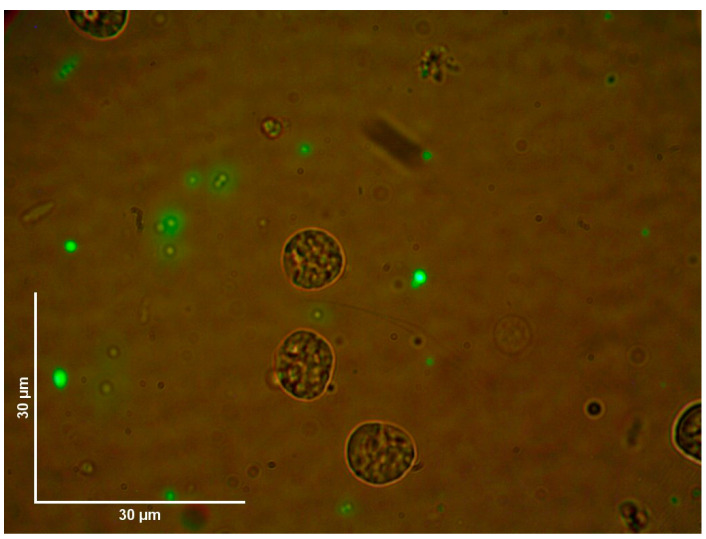
Fluorescence microscopy image of *Rhipicephalus microplus* hemolymph. Images of tissue samples from the tick group treated with 1 × 10^8^ UFC/mL. *S. shinii* S-1 at 2 h posttreatment were obtained via an epifluorescence microscope. Images were captured using white and FITC filters at 1000× magnification.

No fluorescent-stained *E. coli* Top10 was detected in any dissected organ or hemolymph in the treated or untreated control groups (Figure 3). In contrast, fluorescent *Staphylococcus* bacteria were identified in various organs, including the Gené’s organ, and the vestibular vagina, as early as two hours after treatment (Figure 4 and Figure 5). Epifluorescence microscopic observation of tick tissues at 24 h posttreatment revealed the presence of stained *S. shinii* S-1 and *S. succinus* in the salivary glands. Stained *S. succinus* was observed in Gene’s organ (Figure 4) and in the vestibular vagina at 24 h (Figure 5). After 48 h, stained *S. succinus* and *S. shinii* S-1 were observed in the salivary glands and vestibular vagina. Salivary glands infected acini with *S. succinus* and *S. shinii* S-1 appeared to have been damaged during infection (Figure 4), and stained bacteria were found in the hemolymph as early as two hours post-infection (Figure 6 and Figure 7).

## 4. Discussion

The cattle tick is known for transmitting harmful bacteria and blood parasites to bovines. However, few studies have focused on the full cattle tick-associated microbiome, particularly the role of individual microbial species that may serve as entomopathogens. Some of these few reports have identified *Staphylococcus* sp. bacteria within *R. microplus* soft tissue [27], including the tick salivary glands, through microbiome analysis [26]. A previous study using an adult immersion test found that the *Staphylococcus* strains used in this work (*S. shinii* S1, *S. succinus*) have a harmful effect on ticks. *S. shinni* S1 led to a 47% reduction in tick reproduction, 27.5% mortality, 12% less egg laying, and 39.8% reduced hatching. *S. succinus* caused a 44% reduction in tick reproduction, 16% mortality, 3.11% less egg laying, and 44% reduced hatching [14]. This study aimed to determine the localization and distribution of these bacteria within the tick post-exposure. During this study, we located *S. shinii* and *S. succinus* entomopathogenic bacteria within the salivary glands 24 and 48 h after experimental bacterial exposure in engorged females (Figure 4); however, we could not detect any fluorescent bacteria in the gut, suggesting that the experimental bacteria could not penetrate the gut during our experiment. Alternatively, it is possible that the fluorescence was quenched by the presence of blood cells, as previously documented [35], which could have masked the detection of the bacteria. Further discussion and investigations are needed to understand these observations fully. The *Staphylococcus* found in the gut through microbiome analysis in previous studies likely represent different species that are part of the normal tick microbiome [26,27,29] rather than the specific acaropathogenic bacteria under investigation in our study. Pathogens that use ticks as vectors must travel across the gut wall; this event is crucial in completing their life cycle [18,19,20,21,25]. This distinction may explain the differences between the absence of the intestinal location of acaropathogenic microorganisms and the obligate intestinal presence of tick-borne pathogens, which depend on the completion of their life cycle.

We observed a time frame of 48 h for our experimental analysis, which can be considered the initial stage of tick bacterial infection. However, the optimal time for the *Staphylococcus* bacteria to exhibit their acaropathogenic activity on ticks remains to be determined. This study focuses on the final life cycle stage of ticks. However, investigating the impact of *Staphylococcus* bacterial infection on ticks during larval and other free-living developmental stages is crucial for biological control. A loss of larval viability has been reported when this developmental stage is exposed to bacteria through a modified larval package test bioassay [14]. It remains to be determined whether the larvae salivary glands are affected at this early stage of development. This raises questions about the impact of *Staphylococcus* bacterial infection on the feeding and reproduction of ticks. Fluorescent bacteria were identified in the vestibular vagina of engorged female ticks when they were experimentally exposed to *S. succinus* as early as two hours posttreatment, indicating rapid infection by this bacterial species (Figure 5). In contrast, for *S. shinii* S-1, fluorescent bacteria were observed only 24 h posttreatment in the same organ (Figure 5).

These findings suggest distinct tick infection routes for different *Staphylococcus* bacteria species. Previous reports suggest that an indicator of *Staphylococcus* infection in ticks is the presence of exudate around the genital pore or the hypostome [9,14] (Figure 1). During our study, we observed a reduction in tick oviposition attributed to *Staphylococcus* infection in the engorged female’s vestibular vagina, which may obstruct the passage of eggs through the oviduct and effectively reduce oviposition. This obstruction is likely due to blockage of the genital pore by the bacterial exudate previously reported [9] and the consequent swelling of the tick [9]. The rapid vaginal infection observed with *S. succinus*, compared with the delayed infection with *S. shinii* S-1, could explain the more significant proportion of ticks exhibiting swelling after exposure to *S. succinus* than after exposure to *S. shinii*-SI, as previously reported [14]. No stained bacteria were found in either the oviduct or the ovary, indicating that these bacteria may require more time to migrate to these structures. Additionally, some degree of contraction of the vestibular vagina was observed in the *S. shinii* S-1-treated group.

Additionally, fluorescent bacteria were detected in Gené’s organ at 24 h posttreatment, but there was no detection at 48 h posttreatment; this was observed consistently in all the dissected ticks, suggesting that the tick could control the infection. This is likely due to the antimicrobial wax-producing capacity of the Gené’s organ [36,37]. Fluorescent bacteria were consistently detected near organs, such as the trachea and synganglion, but not within those organs. On the other hand, fluorescent bacteria were consistently detected in the hemolymph just two hours after infection (Figure 3 and Figure 4). These findings suggest that *Staphylococcus* entomopathogenic bacteria may use the hemolymph as a pathway to reach other organs. This pattern of bacterial transmission appears similar to that observed in other bacteria or protozoa transmitted by tick vectors [17,38,39]. The presence of bacteria in specific organs and at various times is illustrated in Figure 6.

**Figure 8:**
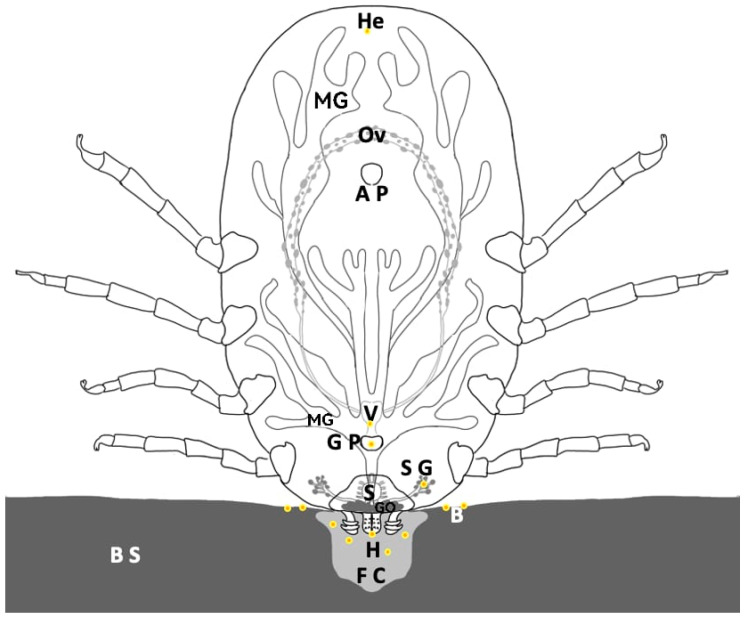
Pathways of introducing skin origin bacterial infection in ticks during blood feeding. Skin bacteria may infect a tick’s internal organs via the following mechanism: The tick attaches to the bovine skin (B S) by introducing a hypostome (H). It induces the formation of a feeding cavity (F C) within the skin colonized by skin bacteria (B). During the blood-feeding process of the tick, the bacteria contained in the blood invade different organs, including the hemolymph (He), salivary glands (S G), Gené’s organ (GO), vagina (V), and genital pore (G P), according to our experimental data. No evidence of infection of the midgut (MG), ovary (Ov), synganglion (S), or anal pore (A P) was found during this experimental study.

Before the tick’s infection, the bacteria source remains uncertain; however, previous reports indicate that this particular *Staphylococcus* species constitutes part of the normal microbiota on the bovine skin [14]. Considering these previous reports and the data supported by our study, we propose that *R. microplus* tick *Staphylococcus* bacterial infection is acquired during the blood-feeding process. In this proposed scenario, once the tick inserts the hypostome through the bovine’s skin, a feeding cavity forms around it (Figure 8). This cavity may serve as a culture medium for the multiplication of *Staphylococcus*, which is part of the normal microbiota of bovine skin, potentially contaminating the blood supply during feeding. The feeding process involves alternating between salivation within the feeding cavity and blood sucking, which includes the periodic opening and closing of the pharyngeal valve. During these processes, bacteria traveling in the blood may reach either the salivarium or continue to the pharynx, esophagus, and midgut.

Our results revealed the presence of bacteria in the hemolymph, salivary glands, and vestibular vagina, indicating that the bacteria might migrate from the salivarium to other tissues, such as the salivary glands, hemolymph, and vestibular vagina [40]. This pathway might also involve Gené’s organ in the case of *S. succinus* infection. Unless our experimental methods fail to detect bacteria in the midgut, it seems likely that the bacteria enter the midgut of the tick and subsequently travel through the hemolymph to reach other tissues, such as the vestibular vagina and salivary glands. This migration pattern aligns with those observed in various well-known bacteria and protozoans that serve as tick-borne pathogens [18,19,20,23,25]. For *S. succinus*, we detected bacteria in the vestibular vagina as early as two hours posttreatment, suggesting a potentially more direct route of infection, such as through the genital pore, especially when the exudate exiting the hypostome reaches the near genital pore. As with other pathogen species, vertical bacterial transmission cannot be ruled out. Previous studies reported that tick larvae hatched from eggs laid by otherwise healthy engorged female ticks carried *Staphylococcus* bacteria [9]. The larvae hatched under laboratory-controlled conditions, suggesting that the only plausible source of *S. saprophyticus* bacteria was their apparently healthy mothers. This finding aligns with recent studies that suggest that maternal transmission of microbiota to eggs can occur during their passage through the genital pore exudate [41,42,43]. In our case, the presence of bacteria in the vestibular vagina, through which the eggs passed during oviposition, further supports this theory.

## 5. Conclusions

In this work, we investigated the in situ localization of *Staphylococcus shinii* and *Staphylococcus succinus* in infected *Rhipicephalus microplus*. These bacteria have previously been demonstrated to exhibit acaropathogenic activity against *R. microplus* and are also components of the bovine skin microbiome. Our study revealed that after exposure, these bacteria can infiltrate the hemolymph and infect areas such as the salivary glands, Gené’s organ, and vestibular vagina of ticks. This infection correlates with observable symptoms such as swelling and reduced oviposition.

These findings suggest that these bacteria could be explored as biological control agents during cattle tick infestations, offering an alternative to chemical pesticides, particularly in regions facing pesticide resistance challenges. These bacteria are part of the natural bovine skin microbiome, so they are expected to pose minimal risk to cattle if applied directly to the skin. However, further research is necessary to address potential impacts on animal and human health when these bacteria are used for biocontrol purposes.

Additionally, these bacteria offer a valuable opportunity to understand better the interactions between bacteria and ticks, including why some bacteria become acaropathogenic and how this trait might be harnessed for tick control. Continued exploration in this area could lead to innovative strategies for managing tick populations more effectively and sustainably.

## Data Availability

Data are contained within the article.
